# The potential of health literacy to address the health related UN sustainable development goal 3 (SDG3) in Nepal: a rapid review

**DOI:** 10.1186/s12913-017-2183-6

**Published:** 2017-03-27

**Authors:** Shyam Sundar Budhathoki, Paras K. Pokharel, Suvajee Good, Sajani Limbu, Meika Bhattachan, Richard H. Osborne

**Affiliations:** 1 School of Public Health & Community Medicine, B P Koirala Institute of Health Sciences, Dharan, 56700 Nepal; 2 World Health Organization, South-East Asia Regional Office, New Delhi, India; 3 Health Systems Improvement Unit, Centre for Population Health Research, Faculty of Health, Deakin University, Burwood, Australia

**Keywords:** Health literacy, Health system responsiveness, Impediments to public health in Nepal, Development, Nepal, Sustainable development goals, SDG, Equity

## Abstract

**Background:**

Health literacy has been linked to health outcomes across population groups around the world. Nepal, a low income country, experiences the double burden of highly prevalent communicable as well as non-communicable diseases. The World Health Organization (WHO) has positioned health literacy as a key mechanism to meet the health-related Sustainable Development Goal (SDG3). However, there is little known about the status of health literacy in developing countries such as Nepal. This paper aims to review the potential of health literacy to address SDG3 in Nepal.

**Methods:**

A rapid review was conducted using the knowledge to action evidence summary approach. Articles included in the review were those reporting on barriers to health care engagements in Nepal published in English language between January 2000 and December 2015.

**Results:**

Barriers for healthcare engagement included knowledge and education as strong factors, followed by culture, gender roles, quality of service and cost of services. These barriers influence the Nepalese community to access and engage with services, and make and enact healthcare decisions, not only at the individual level but at the family level. These factors are directly linked to health literacy. Health literacy is a pivotal determinant of understanding, accessing and using health information and health services, it is important that the health literacy needs of the people be addressed.

**Conclusion:**

Locally identified and developed health literacy interventions may provide opportunities for systematic improvements in health to address impediments to healthcare in Nepal. Further research on health literacy and implementation of health literacy interventions may help reduce inequalities and increase the responsiveness of health systems which could potentially facilitate Nepal to meet the sustainable development goals. While there is currently little in place for health literacy to impact on the SDG3, this paper generates insights into health literacy’s potential role.

## Background

Health Literacy is defined as “the cognitive and social skills which determine the motivation and ability of individuals to gain access to, understand and use information in ways which promote and maintain good health” [1]. It incorporates the characteristics of an individual along with the supports needed to access, understand, appraise and use the information and services to make decisions about their health and the health of their family and the community [2].

Low health literacy is associated with inadequate knowledge about the health as well as the healthcare system, poor access and utilisation of health services and also increased hospitalization. This leads to poor health outcomes and health inequalities [3–6]. Dimensions of health literacy include cognitive, affective, social and personal skills and attributes [7, 8]. A comprehensive understanding of health literacy is essential to understand the full range of needs of members of the community in order to provide accessible and equitable services to all [2]. Furthermore, having an understanding of the health literacy needs of individuals and communities provides the opportunity to develop interventions to improve health outcomes and reduce inequalities [9, 10].

### Nepal- a low income country with substantial health and development challenges

Nepal is a low income country, ranking seventh among the eight South Asian countries and 147 of 187 countries in the world [11]. The life expectancy at birth in Nepal is 68 years [12]. The country’s population is 26.4 million, with 83% living in rural areas [13, 14]. One fourth of the population lives below the poverty line [15] and the adult literacy rate is 66% however the literacy rate in females is lower at 57% [13].

The doctor to population ratio in Nepal is 0.37/1,000 people (as low as 0.008 in rural areas and 1.5/1,000 people in the capital city). Individuals bear 55% of total healthcare expenditure as out-of-pocket payments [16]. About two thirds of healthcare in the acute sector is provided by private hospitals [17]. Gaps to address the social determinants of health exist in Nepal. While Nepal still faces a burden of infectious diseases struggling with inadequate basic hygiene and sanitation along with deep rooted cultural beliefs, the burden of non-communicable diseases is also on the rise [18, 19]. Limited research has been found mentioning health literacy in Nepal [20–22] and level of health literacy of the people of Nepal is not known.

### Health-related sustainable development Goal 3 (SDG3)

While SDG3 *Ensure healthy lives and to promote wellbeing at all ages* is the only specific health goal among the SDGs, other goals, e.g., SDG1 (*No poverty*), SDG2 (*Zero hunger*), SDG4 (*Quality education*), SDG8 (*Decent work and economic growth*), and SDG10 (*Reduced inequalities*) are linked to health and will contribute to improvement of overall population health. SDG3 addresses maternal health, neonatal and child health, AIDS, tuberculosis, malaria and includes universal access to sexual and reproductive health services including family planning. Nepal made progress with the Millennium Development Goals through improvements in maternal and child health. With these achievements, Nepal, like all other countries, is to now set to work towards achieving the SDGs by 2030.

At the 9th Global Conference on Health Promotion 2016, the World Health Organization (WHO) launched the Shanghai Declaration where health literacy is positioned as a foundation block for health and sustainable development in the coming decades [23]. However previous research in Nepal has paid little attention to health literacy, that is, how people and the community might be empowered to engage in recognizing health needs, how to improve knowledge about the health system, and enabling people to regard access to health services as a right. These individual and community attributes are critical components of health literacy and it is critical to understand these such that health literacy can be used to assist with overcoming such impediments and strengthen the health system, improve health outcomes, and, ultimately, to meet the SDG3 in Nepal. This paper discusses the potential of health literacy to address the known and potential impediments for health in Nepal to meet the health-related SDG3.

## Methods

### Study design

A rapid review was conducted using the knowledge to action evidence summary approach [24]. The review question was: What are the impediments of public health in Nepal that could potentially be addressed by health literacy?

### Search strategy

The key words used were taken from the WHO SEARO Health Literacy Toolkit [2] as: ‘access’ , ‘appraise’ , ‘understand’ , ‘decide’ , ‘availability’ , ‘accessibility’ , ‘healthcare’ , ‘utilisation’ , ‘health service’ , ‘ability to decide’ , ‘decision making in health’ , ‘willingness to engage’ , ‘health system responsiveness’ in different combinations with ‘impediments’ and ‘barriers’; with ‘health’ and ‘Nepal’. We limited our search engines to Pubmed, Google Scholar and Nepal Journal Online.

### Inclusion and exclusion criteria

**Fig. 1:**
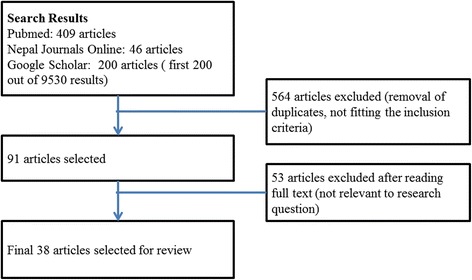
Article selection process

The inclusion criteria were set to include all articles published between January 2000 and December 2015 and reporting on factors influencing healthcare seeking and utilization in Nepal. Articles not published in English were excluded. All articles identified in the search were subjected to the filtering process as shown in Fig. 1.

### Theoretical framework

While many barriers were identified that influenced geographical accessibility to healthcare [25], we used a conceptual framework to categorize barriers for engagement in healthcare (income and price, culture and gender, knowledge and education, and quality of services) as adapted from the access to healthcare in developing countries model by O’Donnel [26].

### Data extraction

Two reviewers independently performed title, abstract and content analysis for the matching the inclusion criteria. A data extraction form was used to record the factors influencing healthcare and disagreement between the two researchers were agreed upon through consensus by the whole research team.

### Synthesis of review

**Fig. 2:**
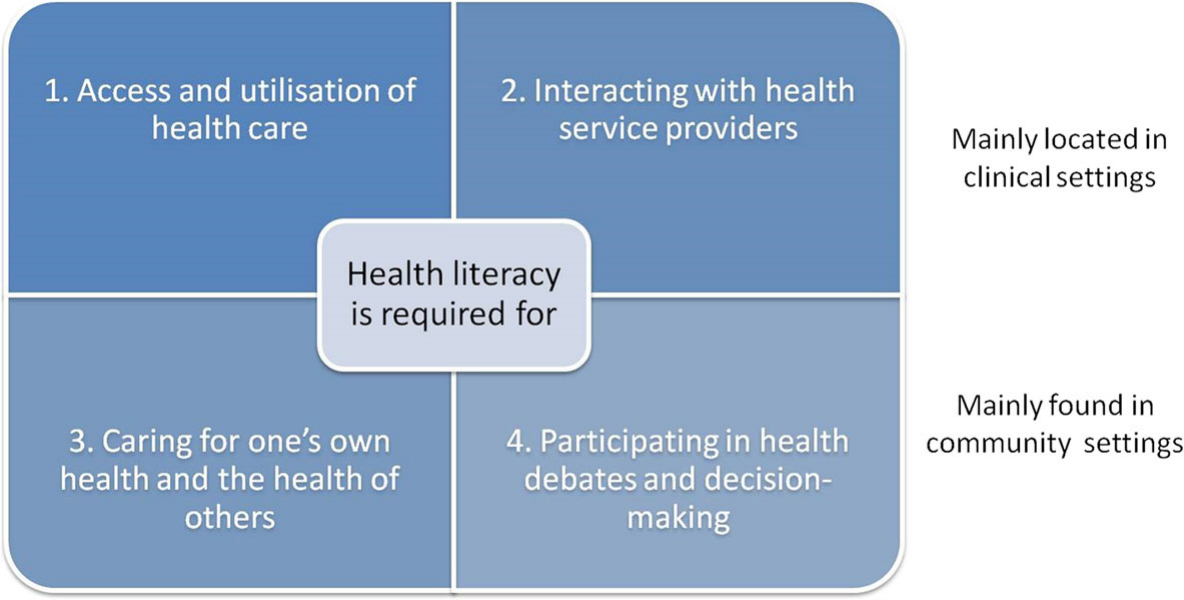
Causal pathway for health literacy influencing health outcome [9]

The identified factors were then classified using four categories; income and price, culture and gender, knowledge and education, and quality of services. To further organize the literature to reveal potential levels of health literacy action we used the four causal paths described by Batterham et al. (Fig. 2) [9].

### Quality assessment

The Assessment, Development and Evaluation (GRADE) approach [27] was used to assess the quality of evidence for trials, case control, cross-sectional and qualitative studies and the AMSTAR checklist was used to assess the quality of the reviews [28]. The articles extracted were independently assessed for quality by two reviewers and if a disagreement occurred a third team member undertook an addition review and negotiated a consensus.

## Results

Overall, 38 original articles included in the review covered a variety of factors influencing healthcare service utilisation (Table 1). There are 5 review articles, 1 trial, 1 case control, 24 cross-sectional and 7 qualitative studies included in this review. The review articles included are of either medium or low quality as classified by the AMSTAR checklist. The trial was of medium quality and the observational studies were either low quality or very low quality as classified using the GRADE approach.

**Table 1:** Factors influencing healthcare service utilisation in Nepal

S N	Author/Year	Objectives	Type of study	Sample size	Relevant findings
1	(Acharya 2010) [52]	To establish the most important socio-background characteristics associated with women’s decision-making power	Cross sectional	8257 married women	Women with high education level have greater autonomy in the decision making for their own healthcare.
2	(Allendorf 2007a) [37]	To comparing spouses’ reports of women’s autonomy with health outcomes in Nepal.	Cross sectional	1858 currently married couples	Women with higher autonomy for household decisions have more access to healthcare.
3	(Allendorf 2007b) [42]	To explore the connections among women’s land rights, women’s empowerment, and child health in Nepal	Cross sectional	4884 women	Children of women with decision making power in the family were less undernourished.
4	Atteraya 2010 [41]	To examine the relationship between women’s autonomy and ability to negotiate safer sex practices among married women.	Cross sectional	8896 married women	Women with higher autonomy in household decision making could also negotiate safe sex.
5	Baral 2010 [63]	To identify the issues associated with women’s role and choices regarding use of Skilled Birth Attendants and to explore factors affecting utilisation of maternal health services in Nepal.	Review	Number of papers not mentioned	Availability of transportation and distance to the health facility, lack of infrastructure and services, availability and accessibility of the services, healthcare cost; inadequate staff, women’s status in the society; women’s involvement in decision making; contribute to utilisation of Skilled Birth Attendance for delivery.
6	Baral 2012 [33]	To identify the range and pattern of maternal health service utilisation in Nepal over	Review	Number of papers not mentioned	Women with higher education and living in urban areas are more likely to use maternal health services.
7	Bhatta 2015 [30]	To assess associated paternal factors and degree of inequity in access to maternal healthcare service utilization.	Cross sectional	2200 men	Husbands with higher education and higher income facilitate their wives to make ANC visits and institutional delivery.
8	Bhattarai 2015 [47]	To explore health seeking behavior and utilization of healthcare services in the rural places in VDCs of Ilam district	Cross sectional	300 men and women	People seek healthcare from traditional healers due to perception of high cost in modern medicine. Private institutions are preferred compared to the public.
9	Bhusal 2011 [48]	To find out the effectiveness and efficiency of Aama Surakshya Karyakram to address barrier in accessing maternal health services in Nepal.	Cross Sectional	47 women	Pregnant mothers were not aware of the provision of incentive for institutional delivery. Of those who were aware, did not know what the incentive was for. Financial incentives are seen to increase the utilisation of maternal health services.
		Methods:			
10	Budhathoki 2014 [60]	To find the factors associated with awareness of occupational hazards and protective measures and the use of protective measures, and the possible relationship between awareness and actual use of PPE.	Cross sectional	300 welders	Welders with higher education are more aware of the hazards and utilise more protective measures.
11	Brunson 2010 [55]	To identify impediments to receiving obstetric care in a context where the infrastructure and services were in place. This	Qualitative	30 women	Women are not aware about the general danger signs of pregnancy, which in turn hinders timely seeking of pregnancy care.
12	Byrne 2013 [65]	To identify demand-side barriers to the utiliza- tion of formal RMNCH services in the Mountains ecological region of Nepal	Review	23 papers	Low status of women, caste discrimination, less knowledge of healthcare, less active mothers groups, dissatisfaction quality of care, health worker attitudes and cultural/spiritual traditions affect healthcare utilisation.
13	Chapagain 2006 [39]	To appraise conjugal power relations and explore the nexus between such relations and couples’ participation in reproductive health (RH) decision-making.	Cross sectional	223 married couples	Gender power relations, traditional gender roles and cost associated with service affects reproductive healthcare decision making.
14	Choulagain 2013 [56]	To examine the characteristics associated with utilization of SBA services in mid- and far-western Nepal	Cross Sectional	2,481 women	Women’s awareness of danger signs of pregnancy, distance from health facilities and inadequate transportation pose major barriers to the utilisation of skill birth attendants’ services.
15	Furuta 2006 [36]	To examine the influence of four indica- tors of women’s household position on the receipt of skilled antenatal and delivery care: their involvement in decision making about their own healthcare and about large household purchases, their employment and control over their own earnings, and their discussion of family planning with their husbands.	Cross sectional	4,695 currently married women	Women supported by husbands, women with higher education were more likely to seek maternal healthcare.
16	Ghimire 2009 [64]	To identify the barriers of access to sexual health services by FSWs in Nepal	Mixed-method	425 female sex workers (FSWs) for quantitative survey and 15 FSWs for in-depth interview	Lack of confidentiality, discriminatory attitudes by healthcare providers, communication gap with service providers and fear of public identification as a sex worker were barriers in seeking sexual health services by the female sex workers.
17	Gubhaju 2009 [54]	To provide indepth examination of the link between husbands’ and wives’ education levels and method of choice of family planning.	Cross Sectional	21,057 women	Level of education of husband and wives affects the choice of family planning method adopted by women.
18	Halim 2011 [51]	To examine the correlates and consequences of antenatal care utilization in Nepal	Cross sectional	3,549 mothers and 2,460 children (0–36 months)	Maternal & paternal education play important role in the utilisation of routine antenatal care.
19	Hotchkiss 2001 [25]	To assess the impact of this investment on the use of maternal healthcare services.	Cross Sectional	1,434 women of reproductive age	Physical access to a healthcare facility affects the utilisation of maternal health services.
20	Iriyama 2007 [59]	To examine the associations between two subscales, perceived severity and perceived susceptibility, and the abstinence intentions of male adolescent students in Nepal.	Cross sectional	297 male students	Knowledge of HIV AIDS among adolescents affected their sex behavior.
21	Jahn 2000 [66]	To assess the performance of maternity care and its specific service components (preventive interventions in antenatal care, antenatal screening, referral, obstetric care) in Banke District, Nepal	Cross Sectional	136 pregnant women, 146 postnatal women	Availability of comprehensive maternal healthcare was associated with higher utilisation of the services.
22	Mishra 2005 [29]	To analyse the contribution of socio- economic status to non-adherence to DOTS.	Case–control	50 cases of tuberculosis and 100 controls	High travel cost to reach the treatment facility, low socioeconomic status affects non-adherence to anti-tuberculosis treatment.
23	Mullany 2006 [44]	To understand the barriers to male involvement in maternal health and explore men’s, women’s, and providers’ attitudes towards the promotion of male involvement in antenatal care and maternal health.	Qualitative	31 couples and 9 women	Low levels of knowledge are associated with less involvement of males in maternal healthcare of their wives.
24	Mullany 2007 [40]	To test the impact of involving male partners in antenatal health education on maternal healthcare utili- zation and birth preparedness in urban Nepal	Randomised controlled trial	442 antenatal women	Women who received education with their husbands have better birth preparedness.
25	Mullany 2005 [45]	To investigate patterns of household decision-making and the context of male involvement behaviors in Katmandu, Nepal	Cross sectional	592 pregnant women	Good communication between husband and wife leads to increased involvement of husband in maternal healthcare.
26	Onta 2014 [57]	To explore the perceptions of service users and providers regarding barriers to skilled birth care	Qualitative	12 FGDs (7–10 women per group) & 12 FGDs (7–10 ANC service providers)	Inadequate knowledge of services, distance to health facilities, unavailability of transportation, and poor availability of skilled birth attendants, poor infrastructure, less service coverage, inadequate awareness about services/facilities, cultural practices and beliefs, and low prioritization of birth care are barriers to maternal healthcare.
27	Pokhrel 2004 [31]	To map out a hierarchical scale of household decision-making regarding child healthcare.	Cross sectional	8,112 adults	Household income and mother’s education is associated with healthcare seeking for children.
28	Poudel 2015 [50]	To find the existing knowledge gap about the economic burden of HIV/AIDS at the household level in Nepal	Review	7 papers	Lack of awareness of potential economic burden of HIV/AIDS upon household exists in the community.
29	Poudel 2004 [62]	To identify Nepali migrants’ vulnerability to HIV/STIs, and to explore the possible role of migration in causing the HIV/STI epidemic in far western Nepal.	Qualitative	60 migrants	Low knowledge on and low perceived vulnerability to HIV/STIs led to risky behaviour among migrants.
30	Powell-Jackson 2009 [49]	To explore early implementation of the programme at the district-level to understand the factors that have contributed to its low uptake	Qualitative	55 key informants from district health service	Bureaucratic delays in the disbursement of funds, gaps in policy communication to implementers and prople affects utilisation of safe delivery services.
31	Puri 2006 [61]	To analyze the sexual behavior, perceived risk of contracting STIs and HIV/AIDS, and protective behaviors of migrant workers	Cross sectional	1,050 factory workers	Migrant workers are not aware about the consequences of unsafe sex and transmission of HIV.
32	Regmi 2010 [76]	To explore the barriers to using sexual health services, including condom-use among young people in Nepal	Qualitative	50 youth for FGD and 31 in depth interviews	Poor sexual and reproductive health knowledge is a barrier in utilisation of sexual health services among the young people
33	Shah 2015 [38]	To identify the socio-demographic, socio-cultural, and health service-related factors influencing institutional delivery uptake in rural areas of Chitwan district,	Cross sectional	673 women	Role of the husband, role of wife in household decision making, access to material resources, literacy rates, dependency on husband, geographical accessibility, and lack of established transportation infrastructure affects the utilisation of institutional delivery services by women.
34	Sharma 2007 [32]	To examine the association of access to health services and women’s status with utilization of prenatal, delivery, and postnatal care	Cross sectional	3,845 women	Maternal health worker visits, educational status of women, household economic status, number of living children and place of residence are associated with utilisation of maternal health services.
35	Simkhada 2006 [53]	To identify some challenges and suggests way forward in the improvement of maternal health in Nepal.	Review	Number of papers not mentioned	Lack of access to basic maternal healthcare, difficult geographical terrain, poorly developed transportation and communication systems, poverty, illiteracy, women's low status in the society, political conflict, and shortage of healthcare professional are barriers to maternal health in Nepal.
36	Smith-estelle 2003 [46]	To identify isues that affect vulnerability to HIV/STI infection among rural women from migrant communities in Nepal	Cross Sectional	900 ever-married women	Gender discrimination, lack of access to healthcare and education in rural areas, and the precarious economic, legal and social circumstances make the women more vulnerable to HIV/STI.
37	Updhyay 2014 [43]	To determine the perceived influential person on a woman’s decision to utilize antenatal and delivery care services among teen, young adult and adult pregnant women	Cross sectional	315 women	Involvement of husband in family planning decision for healthcare seeking for maternal health services.
38	Witter 2011 [71]	To understand the effects of the policy on health facilities. Study methods included structured forms to retrieve financial and activity data from national, district and facility records	Qualitative	Health managers from 22 health facilities	The utilisation of delivery services is facilitated by availability of free services.

### Income and price

Six studies indicated that cost of services is an important barrier for health service utilisation in Nepal. Low annual income, unemployment and inability to bear travel costs to reach health facilities for treatment were associated with less utilisation of tuberculosis treatment [29]. Household income played a role in illness reporting and subsequent healthcare seeking [30–33]. For children in poorer households, healthcare seeking was postponed until urgent [34].

### Culture and gender

Eleven studies reported on cultural practice and perceived gender roles that influence health seeking behaviour. Gender roles affects illness reporting, healthcare decision making and health expenditure [31]. Healthcare seeking was considered as an investment in the family, however male children were more likely to receive care earlier [31, 35]. Fewer women were involved in household decision making processes. Women who participated in household decision making and those who discussed health issues with their husbands were more likely to use maternal healthcare services [36–39]. Women who received health education along with their husbands were more likely to take care of their own health [40].

Women with higher level of autonomy could negotiate safe sex [41]. Malnutrition was lower in children where their mother had more decision making power [42]. Husbands influenced decisions about care seeking [38, 43–45]. Gender discrimination was seen to increase vulnerability of migrant women for sexually transmitted infections and HIV [46]. Seeking healthcare from traditional healers was common in mountainous regions. The perception of high cost of hospital services was seen as a reason for consulting traditional healers [47].

### Knowledge and education

Among 18 identified studies, pregnant women were less aware of free birthing services [48] including support for transportation to health institutions [49]. There was low awareness of disease as well as the risk of health-related economic burden to the family [50]. Women with higher education were more likely to seek healthcare [32, 33, 36, 38, 51, 52]. Seeking healthcare was less frequent among illiterate women [53]. Educated husbands were more likely to facilitate their wives to visit health facilities [30, 51]. Family planning uptake including the choice of family planning by the women was associated with the husband’s education [54]. Women often had limited understanding of early danger signs and the ways to avoid pregnancy complications [55]. Women who were able to recognise the warning signs of pregnancy complications were more likely to utilise skilled birth attendants (SBA) during deliveries [56]. Increasing awareness among women appeared to increase the uptake of SBA services [57].

Inadequate access to information, as well as services, is a major barrier for young people in the uptake of sexual and reproductive health services [58, 59]. A knowledge to practice gap had been reported in some occupational groups; educated welders were more aware of hazards and more likely to use personal protective equipment [60], and migrant workers, who lack knowledge of diseases were more likely to be engaged in unsafe sex and be exposed to HIV [61], mostly due to low perceived vulnerability [62].

### Quality of services

Among 7 studies, impediments for effective health service delivery were found to be due to poor infrastructure, lack of services, poor communication between health workers and patients, staff shortages and attitudes of clinicians at health institutions that hinder the uptake of services [53, 63]. Low competency of managers to implement programs, delays in disbursement of funds, lack of policy communication among providers and public resulted in suboptimal performance of health programs [49]. Barriers to utilisation of health services were lack of confidentiality, negative attitudes of the healthcare providers and inadequate communication between providers and the patients [64]. Dissatisfaction from service providers’ attitudes and practices lead to under-utilisation of services in a mountainous region [65]. Availability of comprehensive health services was associated with higher utilisation of healthcare [66]. Perceived better quality services in private institutions drove people away from public institutions towards private healthcare institutions [47].

## Discussion

This review has provided an understanding of factors affecting the healthcare engagement by the people of Nepal. These factors are in line with the WHO list of social determinants of health (SDH) [67] that exist as impediments to attain the SDGs. While the SDG3 requires multi-sectoral approach beyond the health sector, addressing the social determinants of health and attaining universal health coverage are essential routes to the attainment of SDG3 [68]. Overall, the most consistent and strongest factor influencing health services utilisation in this review appears to be knowledge and education. Culture and gender roles are also important for Nepal, being a country with 125 ethnic groups and 123 spoken languages [13], with clear evidence of gender inequality which is embedded in local cultures, being linked to health inequality. Measuring health literacy and designing health literacy interventions provides system level solutions to address self-care, disease management and improve system responsiveness in different population groups [9, 69, 70]. Among the identified impediments to public health in this review, health literacy could address social determinants of health that are related to knowledge, education, communication, culture and gender roles and quality of service by empowering people to take care of themselves, families and communities [2].

While income and price factors are likely to be addressed, in part, through universal health coverage, work needs to be done to ensure the population is aware of the services, and that they are free [71]. Public health interventions in Nepal will need to include a focus on improving education, including health education, gender equity with careful consideration of cultural diversity, and strengthening the health system. While literacy of the population is linked with health, research linking health literacy with health outcomes has not yet been undertaken in Nepal. The potential pathways [9] for health literacy to impact on health and equity are different for clinical settings and community settings. Health literacy determinants for factors related to access and communication with healthcare providers are more relevant for clinical settings and the factors related to caring and decision making are more relevant for community settings. See Table 2 for a summary of potential causal pathways for impacting on health and equity in Nepal.

**Table 2:** Summary of potential health literacy-related causal pathways for impacting on health and equity in Nepal

1. Health literacy is required to enable people to access and utilize healthcare	
	People in Nepal have many potential barriers to access and use healthcare services. Barriers include cost of services, cost of transport, low income and unemployment. Existing gender roles and discrimination related to local culture, knowledge of services and health problems, limited availability of services, low quality services provide large challenges for people to access and utilize services.
	To overcome these barriers, the health literacy of community members needs to be high such that people are empowered to be able to make decisions about healthcare and overcome access barriers.
2. Health literacy is required to enable people to have high quality interactions with health service providers	
	Many barriers to quality interactions were identified, including: local culture and gender norms, education, knowledge of health services and health problems, access to good quality information, communication skills of staff, health worker’s attitudes and organizational policy on communication with community members.
	When there are one or more of these potential barriers to quality interaction with health service providers, the health literacy of a community member will need to be high.
3. Health literacy is required to optimize caring for one’s own health and the health of others	
	The identified determinants of this area included gender roles and women’s autonomy, spousal support along with knowledge and education.
	Improving health literacy increases understanding of health and disease as well as the available services, hence people are able to take decisions to take care of their own self and others.
4. Health literacy is required to enable participation in health negotiations and decision-making	
	The review identified few determinants of participation including the ability to engage in discussions related to gender roles and discrimination, women involvement in decision making, men’s involvement in women’s health, women’s autonomy, spousal support, knowledge and education and the health system responsiveness including communication skills of staff and the quality of health services. Another relevant ability in the community level is the ability of an individual to be able to discuss health matters and make decisions about health. This requires adequate health literacy in an individual and across a community. A strong background mechanism is likely to be educational attainment, including having an understanding of basic biomedical concepts including anatomy and basic medical terms. Without these, being empowered to participate in health negotiations and decision making is unlikely.

### Relationship between factors influencing public health, SDGs and health literacy

In this review we only focused on health-related SDG3. However, there are clear links between health literacy and SDG1 (*No poverty*), SDG2 (*Zero hunger*), SDG4 (*Quality education*), SDG8 (*Decent work and economic growth*), SDG9 (*Industry*, *information and infrastructure*), SDG10 (*Reduced inequalities*) and SDG16 (*Peace*, *justice and strong institutions*) [23]. The areas for health literacy interventions identified are likely to have impact on these as they are in line with the causal pathways identified by Batterham et al. [9]. Framing the interventions in this way is useful because it identifies starting points for programmatic interventions. In *health service settings* the focus may be more on the health literacy strengths and limitations of individuals seeking care, the levels of engagement they are able to have with the services, and the ways in which health services can accommodate this diversity, including how services ensure all eligible individuals gain equitable access to the services they provide. Improved health literacy can enhance doctor-patient communication by patients making more informed choices and doctors communicating in plain language to increase the patients’ understanding of their health [72]. Health literacy responsive healthcare professionals can also contribute to improving health literacy of patients by responding to the patients based on their health literacy levels.

At the *community level*, health literacy has many implications regarding daily decisions about health promotion and disease management, not only at the individual level, but decisions for and by family and community units. Health literacy in this setting also has profound implications for an individual’s and community’s ability to comprehend and engaging in negotiations and decision making about health [9].

Knowledge and education are direct determinants of understanding, analysing and critical appraisal abilities which enable people to be aware of the available services and overall understanding of health and disease. Notwithstanding education, the impact of inadequate income, pervasive inequitable cultural practices and poor quality of care, can make decision making about health extremely challenging. While the level of education attained is deemed important [30, 32, 33, 36, 38, 51–54], a lack in knowledge also exists regarding either availability of services, severity of illnesses and/or vulnerability to diseases [48–50, 56–58, 60–62]. Healthcare practices in households have deep roots in cultural beliefs and gender roles [19] thus a strong education system is required to advance this area. Nepal clearly has work to do to strengthen community level health literacy and this will underpin the attainment of SDG3: *Ensure healthy lives and promote well*-*being for all at all ages*.

Healthcare engagement barriers include actual and perceived barriers such as income and price as demonstrated that once the services are subsidized or made free, uptake is increased [71]. There is potential for catastrophic health expenditure that can happen at the household level [73]. These costs affect the uptake of services which in turn will affect the attainment of SDGs. Quality of care is determined by the technical expertise, communication skills, attitudes and policy communication at local and regional levels, but are also strongly related to education and cultural beliefs. While quality of care is more a reflection of the healthcare system, the education and cultural beliefs also strongly determine healthcare service utilisation. Beliefs and behavior can change in individuals and communities through effective communication alongside provision of appropriate physical infrastructure, equipment, physical distribution of facilities and availability of staff. These are factors that require well planned capital investment by central and regional government authorities. Table 3 outlines the SDG3 targets and the factors identified that may impact on Nepal’s ability to attain the targets. The factors underline the social determinants of health in Nepal at both structural and intermediate levels which are needed to be addressed to attain SDG3. Health literacy interventions have the potential to act on people as well as health system to improve health of the people [2].

**Table 3:** SDG3 - *ensure healthy lives and promote well*-*being for all at all ages*: targets and factors likely to influence attainment of health-related goals

Health related of SDG 3: ensure healthy lives and promote well-being for all at all ages	Factors influencing the attainment of targets
3.1 By 2030, reduce the global maternal mortality ratio to less than 70 per 100,000 live births	Income and Cost
	- Cost of Services
3.2 By 2030, end preventable deaths of newborns and children under 5 years of age, with all countries aiming to reduce neonatal mortality to at least as low as 12 per 1000 live births and under-5 mortality to at least as low as 25 per 1,000 live births	- Cost of Transport
	- Income status
	- Employment status
3.3 By 2030, end the epidemics of AIDS, tuberculosis, malaria and neglected tropical diseases and combat hepatitis, water-borne diseases and other communicable diseases	- Socioeconomic status
	Culture and gender
	- Gender roles/discrimination
3.4 By 2030, reduce by one third premature mortality from non-communicable diseases through prevention and treatment and promote mental health and well-being	- Cultural norms of women involvement in decision making
	- Men’s involvement in women's health
	- Womens' autonomy
3.5 Strengthen the prevention and treatment of substance abuse, including narcotic drug abuse and harmful use of alcohol	- Spousal support
	Knowledge and Education
3.6 By 2020, halve the number of global deaths and injuries from road traffic accidents	- Education status
	- Knowledge of services
3.7 By 2030, ensure universal access to sexual and reproductive health-care services, including for family planning, information and education, and the integration of reproductive health into national strategies and programmes	- Knowledge of health problems
	- Knowledge of hazards
	- Knowledge of Economic burden
3.8 Achieve universal health coverage, including financial risk protection, access to quality essential health-care services and access to safe, effective, quality and affordable essential medicines and vaccines for all	- Access to good quality information
	Quality of services
3.9 By 2030, substantially reduce the number of deaths and illnesses from hazardous chemicals and air, water and soil pollution and contamination	- Health system responsiveness
	- Infrastructure
3.a Strengthen the implementation of the World Health Organization Framework Convention on Tobacco Control in all countries, as appropriate	- Availability of Services
	- Communication skills of staff
3.b Support the research and development of vaccines and medicines for the communicable and non-communicable Diseases that primarily affect developing countries, provide access to affordable essential medicines and vaccines, in accordance with the Doha Declaration on the TRIPS Agreement and Public Health, which affirms the right of developing countries to use to the full extent the provisions in the Agreement on Trade-Related Aspects of Intellectual Property Rights (TRIPS) regarding flexibility to protect public health, and in particular provide access to medicines for all	- Health worker’s attitude
	- Human resources for health
	- Technical/Managerial competence of staff
	- Policy and its implementation
	- Policy communication with people
	- Privacy/Confidentiality
3.c Substantially increase health financing and the recruitment, development, training, and retention of the health workforce in developing countries, especially in the least-developed countries and developing small island states	- Satisfaction regarding healthcare
3.d Strengthen the capacity of all countries, in particular developing countries, for early warning, risk reduction, and management of national and global health risks	

### Interventions to improve health literacy, health and equity

While the health sector, including individual health professionals, are major contributors to improving health literacy of the population, the attainment of SDG3 requires collective efforts from all sectors. While health literacy is identified as a “foundation block” for improving global health by the WHO [23] there has been little discussion about how health literacy can be operationalized at scale to achieve such objectives. The South East Asian Regional Office of the WHO recently published a Health Literacy Toolkit for Low- and Middle-Income Countries, which provides insights into how health literacy can be used to impact on systems, services and policy [2, 74]. While our review identified four health literacy intervention points, a process for moving from problem identification to problem solving is required. The toolkit provides guidance for the development and implementation of interventions to address many determinants of health. The toolkit introduced the term “health literacy responsiveness” i.e., “*the way in which services*, *environments and products make health information and support available and accessible to people with different health literacy strengths and limitations*” [2]. This concept fits well with the findings of our review. For Nepal to make systematic improvements at scale, locally derived and tailored interventions need to be generated and implemented. A promising approach for undertaking this is outlined in the toolkit, i.e., a health literacy-focused approach to community development called, Ophelia (OPtimising HEalth LIteracy and Access) [2, 75]. This type of locally derived intervention approach could be coupled with national health literacy needs assessment using health literacy questionnaires specifically designed to guide intervention development.

### Strengths and limitations of the study

The application of the rapid review approach may have led to omission of some published papers. Furthermore, as we did not focus specifically on interventions, the quality assessment could not be applied in detail to generate an overall quality assessment. Nonetheless, this is the first review of a developing country’s status in regard to health literacy and its capacity to respond to the SDGs. It forms a reasonable baseline for Nepal and may be a good exemplar for other low and middle income countries to use to scope current status and what is required for health literacy capacity development to impact on SDG3 and other SDGs.

## Conclusion

While Nepal has challenges ahead to attain the SDG3, this rapid review provides some insights to promote discussion and planning in support of an effective plan. In a resource-challenged country facing substantial burden of disease, health programs in Nepal are often in competition with other personal, family, community and national priorities where trade-offs need to be made between caring for health and attending to other pressing concerns. Knowledge, awareness, culture, language and communication are among the major barriers for health in Nepal where a comprehensive health literacy approach has potential to contribute in improving the health system. While further research on health literacy is clearly needed, there is an immediate role for health literacy in supporting timely utilisation of health services, strengthening health systems; improving health outcomes and reducing health inequities in Nepal.
